# Pseudotumor Cerebri Syndrome and Essential Thrombocythemia: A Case Report

**DOI:** 10.1002/ccr3.72766

**Published:** 2026-05-26

**Authors:** Fang‐Tzu Chang, Yi‐Ying Chen, Fu‐Yu Lin

**Affiliations:** ^1^ Department of Neurology China Medical University Hospital Taichung Taiwan

**Keywords:** essential thrombocythemia, headache, increased intracranial pressure, pseudotumor cerebri syndrome

## Abstract

Pseudotumor cerebri syndrome (PTCS) is characterized by elevated intracranial pressure in the absence of intracranial mass lesions, structural abnormalities, or infectious conditions. Although the exact pathogenesis of PTCS remains largely elusive, it is increasingly recognized as a multifactorial condition. Identifying secondary contributors is essential for guiding etiology‐specific management and achieving an optimal treatment response. Essential thrombocythemia (ET) is a myeloproliferative neoplasm involving sustained thrombocytosis and an elevated risk of thrombotic complications. We present a case of PTCS associated with ET. In this instance, significant symptomatic relief coincided with the initiation of antiplatelet and cytoreductive therapy. This clinical trajectory suggests a potential synergistic association between ET‐related hematologic changes and the development of PTCS.

## Key Clinical Message

Essential thrombocythemia could be considered one of the potential secondary causes of pseudotumor cerebri syndrome (PTCS). Etiology‐specific intervention for PTCS is crucial for improving prognosis in such cases.

## 1 Introduction

Pseudotumor cerebri syndrome (PTCS) is characterized by increased intracranial pressure with papilledema, normal brain parenchyma on neuroimaging, and normal cerebrospinal fluid (CSF) composition [1]. Most patients with primary PTCS, also called idiopathic intracranial hypertension, are female or have obesity or recent weight gain; however, the underlying pathogenesis remains unclear [2]. A survey of identifiable secondary factors of increased intracranial pressure revealed causes, such as venous sinus thrombosis, medications, and systemic or endocrine disorders; emphasizing these secondary causes is vital for etiology‐specific management [1, 2]. Essential thrombocythemia (ET) is a myeloproliferative neoplasm characterized by a sustained elevation of platelet counts (≥ 450 × 10^9^/L). It is commonly associated with specific driver mutations, most notably *JAK2* V617F (occurring in approximately 50%–60% of cases), followed by *CALR* (about 20%) and *MPL* (about 3%). Approximately 20% of patients are classified as triple‐negative, lacking all three aforementioned mutations. ET is known to increase the risk of thrombotic complications [3]. Currently, ET is not formally recognized as a secondary cause of PTCS [1, 4]. In this paper, we report a case of PTCS with papilledema and concurrent ET, where symptomatic relief coincided with antiplatelet and cytoreductive therapy, suggesting a potential association between these two conditions.

## 2 Case History / Examination

A healthy 26‐year‐old woman presented with episodic blurry vision for 5 months. Regarding her visual symptoms, the patient experienced transient visual obscurations lasting approximately 30 s per episode in either eye, with frequency ranging from once every 2–3 days to 10 times per day. She also reported episodic headache and occasional pulsatile tinnitus. Her medical history was negative for seizure disorders, vitamin A supplementation, acne medications, lead exposure, oral contraceptives, or steroid use.

**FIGURE 1:**
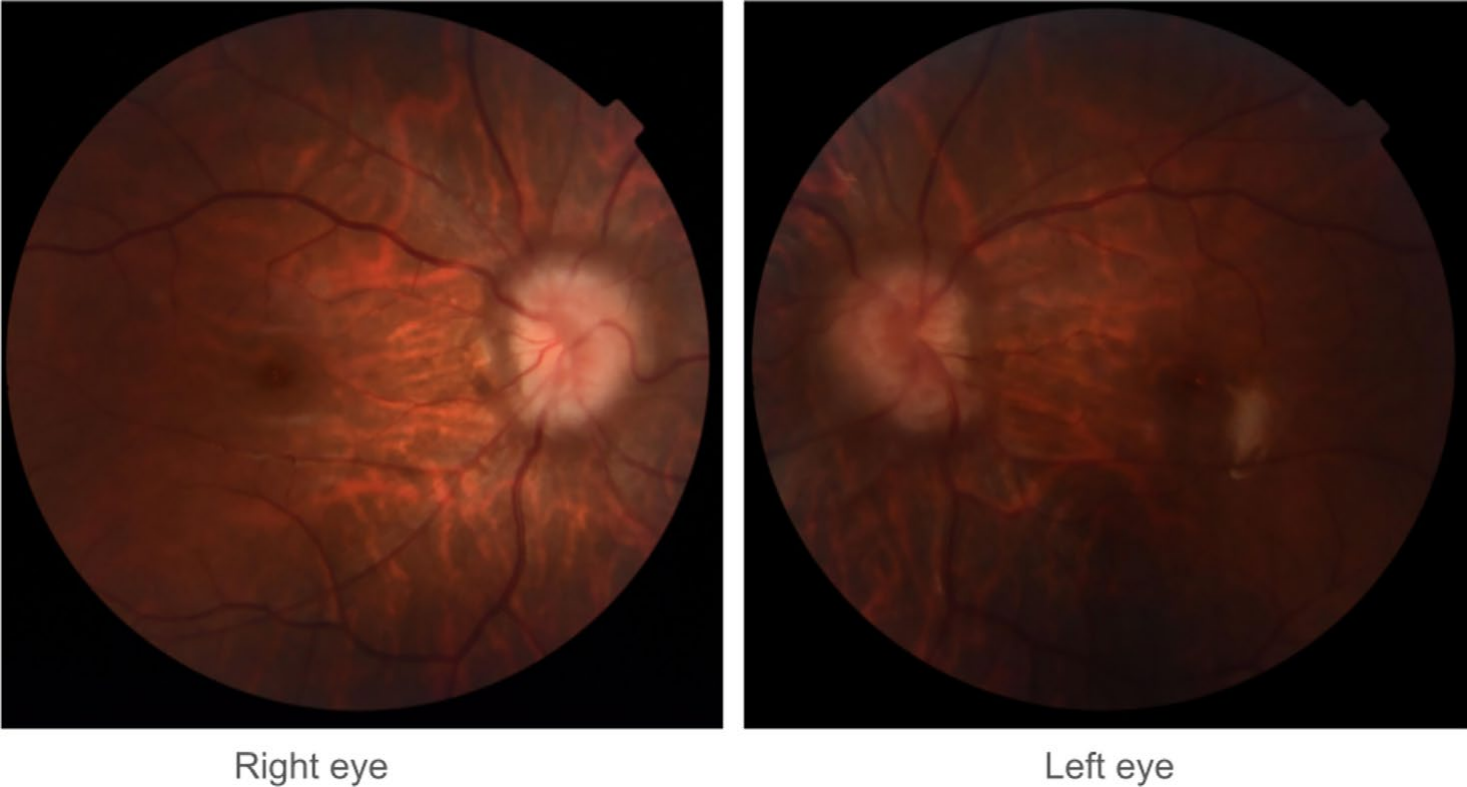
Initial fundus photographs showing bilateral papilledema.

On physical examination, her BMI was 23.6 kg/m^2^. She was conscious and oriented. Her best‐corrected visual acuity was 20/20 in the right eye and 20/25 in the left. Pupillary responses were normal, and there was no relative afferent pupillary defect (RAPD). Intraocular pressure was 17 mmHg in the right eye and 19 mmHg in the left, both within normal limits. Fundus examination revealed bilateral papilledema with an absence of spontaneous venous pulsations (Figure 1). Visual field testing using 30–2 perimetry (Carl Zeiss Meditec Inc., Dublin, CA, USA) showed peripheral field constriction in both eyes, though more prominent on the left. Optical coherence tomography (OCT) with Cirrus HD‐OCT (Carl Zeiss Meditec Inc., Dublin, CA, USA) revealed an average retinal nerve fiber layer (RNFL) thickness of 226 μm in the right eye and 92 μm in the left. Otherwise, the remainder of her neurological examination was unremarkable.

## 3 Differential Diagnosis, Investigations and Treatment

In the present case, the diagnostic workup focused on the etiology of headache associated with increased intracranial pressure (IICP). To ensure patient safety, potential secondary causes of headache, which typically require more urgent intervention, were prioritized. These included vascular pathologies (e.g., aneurysm, arteriovenous malformation, cerebral venous thrombosis, and reversible cerebral vasoconstriction syndrome), infectious etiologies (e.g., viral, bacterial, or fungal meningitis and meningoencephalitis), inflammatory conditions (e.g., vasculitis and multiple sclerosis), mass lesions (e.g., neoplasm, intracranial hemorrhage, and hydrocephalus), and drug‐induced or withdrawal‐related factors (e.g., steroids and retinoic acid). Primary headache disorders, such as migraine and tension‐type headache, were also considered; however, the presence of bilateral papilledema serves as a definitive clinical sign of IICP, necessitating further survey.

Regarding the clinical investigation of this patient, brain magnetic resonance imaging (MRI) with and without contrast yielded unremarkable results. Lumbar puncture revealed a markedly elevated opening pressure of 34.0 cmH_2_O, whereas the cerebrospinal fluid (CSF) composition remained within normal limits. Magnetic resonance venography (MRV) suggested several areas of potential dural venous sinus stenosis, which might be related to intracranial hypertension, whereas digital subtraction angiography (DSA) demonstrated no definitive focal thrombosis or occlusion, but slow flow was noted. The D‐dimer level was within normal limits (244 ng/mL). Serological screening for autoimmune disorders was negative. Laboratory analysis revealed a significant elevation in platelet count (860 × 10^9^/L). The presence of the *JAK2* V617F mutation was confirmed via Allele‐Specific PCR (targeting *JAK2* gene NG_009904.1; 93,497–93,699), following the protocol described by Kralovics et al. [5]. Tests for *CALR* and *MPL* mutations were negative. A definitive bone marrow study was recommended but declined by the patient.

Under the clinical impression of PTCS, initial management involved acetazolamide and topiramate to control intracranial hypertension. However, the patient's visual symptoms and headache were only partially alleviated over a 1‐month treatment course. A subsequent CSF study showed a reduction in opening pressure to 22.0 cmH_2_O.

**FIGURE 2:**
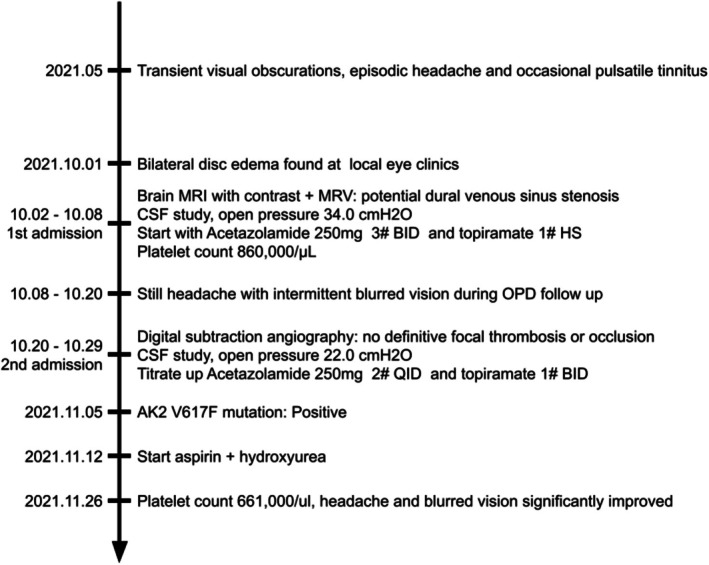
Clinical timeline illustrating the sequence of symptoms, important diagnostic workup and therapeutic interventions.

The initial risk stratification for ET was classified as low‐risk, based on the patient's age and the absence of a confirmed thrombotic event. Nevertheless, the persistence of neurological symptoms despite the reduction in intracranial pressure, coupled with the angiographic evidence of stenosis, prompted a re‐evaluation. To mitigate the risk of potential irreversible ischemic events, we adopted a more intensive management strategy by initiating aspirin and hydroxyurea. This intervention coincided with a significant resolution of her symptoms. Although the exact mechanism remains speculative, this clinical course suggests that ET may have acted as a contributory factor to the development or persistence of PTCS in this patient. (Figure 2).

## 4 Outcome and Follow‐Up

Optical coherence tomography (OCT) was utilized to quantitatively monitor the progression of papilledema throughout the clinical course. Initial assessments documented severe bilateral swelling, which was more pronounced on the right side. After 1 month of conventional therapy with acetazolamide and topiramate, follow‐up OCT revealed partial improvement in the right eye, with an average retinal nerve fiber layer (RNFL) thickness of 191 μm, but worsening edema in the left eye, which showed a significant increase to 170 μm.

**FIGURE 3:**
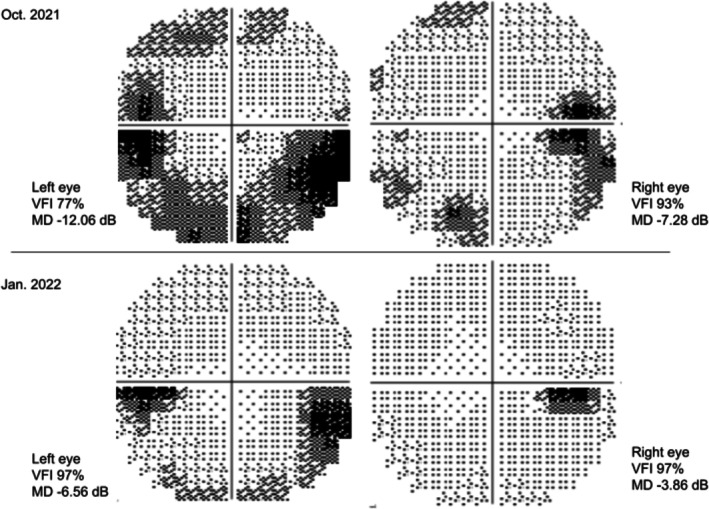
Longitudinal visual field progression. Top: October 2021 (baseline). The visual field index (VFI) was 93% with a mean deviation (MD) of–7.28 dB in the right eye, and 77% with an MD of–12.06 dB in the left eye. The initial visual field demonstrated a significant peripheral constriction pattern. Bottom: January 2022 (3‐month follow‐up). The VFI improved to 97% with an MD of–3.86 dB in the right eye, and 93% with an MD of–6.56 dB in the left eye. The visual field showed a marked reduction in the peripheral constriction pattern compared with baseline.

**FIGURE 4:**
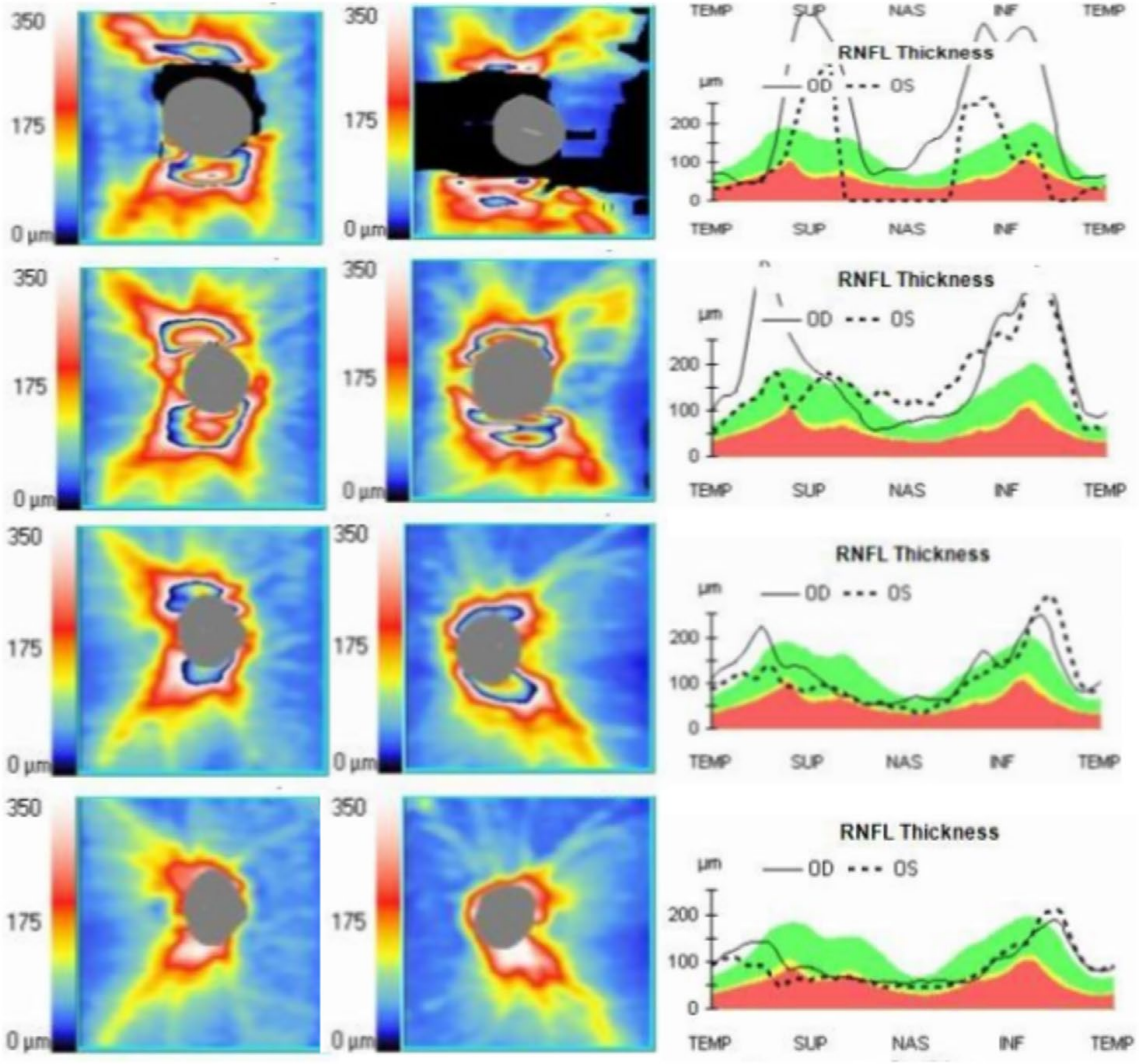
RNFL thickness map. Top to bottom: October 2021 through February 2026. Left panel: right eye; right panel: left eye.

Following the initiation of hydroxyurea and aspirin for ET management, a rapid anatomical response was observed. One and a half months after starting cytoreductive therapy, the average RNFL thickness had markedly decreased to 123 μm in the right eye and 111 μm in the left eye. Complementing the structural improvement, visual field testing at the 3‐month follow‐up demonstrated significant recovery (Figure 3). Long‐term follow‐up, 4 years later, confirmed the complete resolution of papilledema, with RNFL thickness fully normalizing to 97 μm in the right eye and 89 μm in the left eye (Figure 4), (Table 1).

**TABLE 1:** Average RNFL thickness and symmetry corresponding to Figure 4.

Date/Average RNFL Thickness	OD	OS	RNFL Symmetry
October 2021	226 μm	92 μm	83%
Initial presentation			
November 2021	191 μm	170 μm	68%
Post acetazolamide and topiramate			
December 2021	123 μm	111 μm	81%
1.5 months after cytoreductive therapy			
February 2026	97 μm	89 μm	88%
Long‐term follow‐up			

The patient was followed up for 4 years, without recurrence of PTCS.

## 5 Discussion

Patients with PTCS are generally classified into two cohorts: those without an identifiable etiology, termed idiopathic intracranial hypertension (IIH), and those with recognizable secondary causes. Clinically, the presentations of patients with secondary PTCS may be indistinguishable from those with IIH. However, identifying secondary factors is crucial for guiding etiology‐specific intervention [1]. In the present case, although the diagnostic criteria for PTCS were met, initial evaluations found no definitive evidence of common secondary causes, such as cerebral venous sinus thrombosis (CVST). The patient was initially managed for IIH but showed a limited clinical response. Notably, a significant resolution of symptoms was observed after the initiation of aspirin and cytoreductive therapy, suggesting a potential association between ET and PTCS.

The literature describes numerous secondary factors contributing to the development of PTCS, including cerebral venous abnormalities (CVST or arteriovenous fistula), medication exposure (vitamin A, tetracycline, or hormones), and underlying medical conditions (sleep apnea, anemia, or renal failure) [1, 2, 4, 6, 7]. ET, a myeloproliferative neoplasm characterized by a sustained elevation in platelet count, significantly increases the risk of thrombosis [3]. Therefore, it is hypothesized that ET may serve as a potential contributor to PTCS development. Although one study investigated ET patients with headache disorders and ocular ischemic events [8], ET is currently not formally established as an independent risk factor for PTCS.

The pathogenesis of PTCS remains elusive. The diversity of contributing factors suggests that PTCS may not stem from a single underlying cause but rather involves multifactorial pathogenic mechanisms. Furthermore, the fact that not all individuals with these risk factors develop the syndrome underscores its complex nature [7]. Concurrent cases of PTCS and ET are rarely reported, and in the majority of such instances, PTCS was found to be secondary to CVST. However, the absence of CVST in certain cases, including the present one, suggests the possibility that ET might independently contribute to PTCS. One proposed mechanism is that headache and visual symptoms may result from intermittent microvascular disturbances or transient cerebral venous sinus compromise associated with extreme thrombocytosis [9].

Recent molecular evidence suggests that the *JAK2* V617F mutation is linked to the upregulation of pro‐thrombotic genes in platelets and neutrophils, which enhances leukocyte adhesion and the release of neutrophil extracellular traps. Furthermore, endothelial cells harboring the *JAK2* V617F mutation have been shown to secrete increased levels of pro‐thrombotic and adhesive proteins, such as von Willebrand factor and P‐selectin [10]. A case series examining the *JAK2* V617F mutation in patients with PTCS associated with CVST noted that most patients had concomitant thrombocytosis and generally poor visual outcomes despite intervention, warranting a more aggressive management approach in this specific subpopulation [11].

Regarding the selection of cytoreductive therapy, interferon‐based regimens are often preferred over hydroxyurea for younger patients to mitigate potential long‐term leukemogenic risks. However, in our clinical setting, several factors influenced the choice of hydroxyurea. First, the only available interferon formulation in our facility is peginterferon, which is not covered by national health insurance for this indication, posing a significant financial burden. Similarly, anagrelide was considered but is also not currently reimbursed. Despite these constraints, the patient has maintained stable platelet counts and demonstrated a favorable clinical response to hydroxyurea without significant adverse effects. We continue to monitor her hematologic profile closely for any long‐term complications.

We addressed whether the dural venous sinus stenosis identified on imaging warranted the placement of a venous sinus stent. Although venous sinus stenting has emerged as a recognized treatment modality for PTCS associated with stenosis, we prioritized the hematologic management of ET. This decision was informed by the diffuse nature of the patient's vascular findings; imaging revealed multiple potential areas of dural sinus narrowing rather than a focal lesion isolated to a single transverse sinus. The multifocal and widespread distribution of these narrowings led us to hypothesize that the venous sinus stenosis was likely a result of extrinsic compression secondary to the elevated intracranial pressure itself, rather than serving as the primary anatomical driver of hypertension. Consequently, a systemic approach targeting the underlying hematologic condition was deemed more clinically appropriate and less invasive than a localized interventional procedure.

In the present case, the proposed pathophysiological mechanism suggests a vicious cycle of pressure dynamics: although the anatomical venous sinus stenosis could be a secondary consequence of intracranial hypertension, such narrowing may, in turn, further compromise CSF outflow. Additionally, concurrent thrombocytosis potentially exacerbated this equilibrium through increased blood viscosity or microvascular dysfunction, leading to a synergistic elevation in intracranial pressure.

Several limitations of this case report warrant consideration. First, regarding the diagnosis of ET, the absence of bone marrow aspiration and biopsy data meant the patient did not strictly fulfill the complete WHO diagnostic criteria. Despite extensive counseling, the patient's refusal of the procedure remains a significant constraint. To address this, we relied on clinical surrogates—including the absence of anemia, splenomegaly, and leukoerythroblastosis—combined with the presence of the *JAK2* V617F mutation and a sustained high platelet count to support the clinical impression of ET. Furthermore, our facility's current inability to provide the variant allele frequency (VAF) has been noted as an additional limitation.

Second, the observed therapeutic response cannot be exclusively attributed to cytoreductive therapy. Although a single‐case experience cannot be generalized to the broader population, it is noteworthy that complete clinical stabilization was achieved only after the addition of hydroxyurea. Although a delayed therapeutic effect from acetazolamide and topiramate cannot be entirely discounted, the strong temporal correlation suggests that managing the underlying thrombocytosis played a contributory role in resolving the PTCS presentation.

Third, although we captured the clinical progression through serial OCT measurements—including a documented period of suboptimal response to conventional diuretics prior to the initiation of cytoreductive therapy—the retrospective nature of this report meant that these assessments were performed at clinically determined intervals rather than a standardized study protocol. However, the temporal correlation between the introduction of hydroxyurea and the subsequent rapid decline in RNFL thickness provides compelling evidence for the efficacy of our multimodal approach.

In conclusion, this case report highlights a potential association where ET might serve as a secondary contributing factor to PTCS. Early intervention in patients with PTCS is crucial for preventing permanent visual impairment resulting from ischemic optic neuropathy. The management of PTCS patients with concurrent ET may involve the implementation of platelet‐lowering therapies, such as hydroxyurea, alongside antithrombotic therapy. Further studies are warranted to elucidate the precise pathophysiological mechanisms and to develop targeted therapeutic strategies for this specific patient population.

## Author Contributions

**Fang‐Tzu Chang:** validation, writing – review and editing. **Yi‐Ying Chen:** writing – original draft. **Fu‐Yu Lin:** conceptualization, data curation, investigation, methodology, project administration, resources, supervision, writing – original draft, writing – review and editing.

## Funding

The authors have nothing to report.

## Ethics Statement

As a single‐case report with the patient's signed consent, no other ethical review was required.

## Consent

Written informed consent was obtained from the patient for the publication of this case report.

## Conflicts of Interest

The authors declare no conflicts of interest.

## Data Availability

All relevant data are included in this manuscript.
